# Content validity of the ASQoL for use in a non-radiographic axial spondyloarthritis population: a qualitative study

**DOI:** 10.1007/s11136-020-02552-z

**Published:** 2020-07-01

**Authors:** Mark C. Hwang, Mona Martin, Kristina Harris, Philip Geerdts, Jeffrey L. Stark, John Reveille

**Affiliations:** 1 McGovern Medical School, The University of Texas Health Science Center at Houston, Houston, TX USA; 2 Evidera PPD LLC, Bethesda, MD USA; 3 UCB Pharma, Hong Kong, China; 4 UCB Pharma, Slough, UK; 5 UCB Pharma, Smyrna, GA USA; 6 Department of Internal Medicine, McGovern Medical School, The University of Texas Health Science Center at Houston, 6431 Fannin, MSB 1.150, Houston, TX 77030 USA

**Keywords:** Ankylosing spondylitis quality of life, Content relevance, Health-related quality of life, Non-radiographic axial spondyloarthritis, Qualitative interviews

## Abstract

**Purpose:**

The ankylosing spondylitis quality of life (ASQoL) instrument is widely used to assess health-related quality of life in patients with ankylosing spondylitis (AS). We assessed the relevance of the ASQoL items in patients with non-radiographic axial spondyloarthritis (nr-axSpA), a distinct subgroup within the axSpA disease spectrum.

**Methods:**

This observational, cross-sectional, qualitative interview study recruited patients from clinic settings. Interviews from patients with axSpA who participated in a prior qualitative study were also used. Patients initially underwent a concept elicitation interview using open-ended questions to evaluate relevance of the concepts measured by the ASQoL. They then completed the ASQoL and underwent a cognitive interview to assess their understanding of the items, instructions and response options. Transcripts from patients who participated in the previous qualitative study (who did not complete the ASQoL or undergo cognitive interview) were evaluated to identify expressions of the concepts in the ASQoL.

**Results:**

A total of 18 patients with nr-axSpA participated. The concept elicitation interview findings supported the relevance of the ASQoL items. Cognitive interviews determined that the ASQoL was easily understood; the 13 new patients chose a response for each item that matched their experience with nr-axSpA. Transcripts for the five previously interviewed patients confirmed the concepts presented in the ASQoL items were relevant and important to their experience of living with nr-axSpA.

**Conclusions:**

Our results represent an important first step in confirming the relevance of the concepts in the ASQoL to patients with nr-axSpA, supporting quantitative assessment of ASQoL validity in this population.

**Electronic supplementary material:**

The online version of this article (10.1007/s11136-020-02552-z) contains supplementary material, which is available to authorized users.

## Introduction

Axial spondyloarthritis (axSpA) is a chronic inflammatory disease with a heterogeneous clinical phenotype that primarily affects the sacroiliac joints and spine. Patients with axSpA are classified into two subgroups based on the presence or absence of clearly defined sacroiliitis on conventional pelvic X-rays [1, 2]. Patients with ankylosing spondylitis (AS) have definitive radiographic evidence of sacroiliitis (as per modified New York criteria [mNY]) [3]. Patients with non-radiographic axial spondyloarthritis (nr-axSpA) do not show definitive radiographic sacroiliitis but have the typical clinical manifestations of axSpA and often have inflammation of sacroiliac joints on magnetic resonance imaging (MRI).

nr-axSpA may represent an early stage of disease that progresses to AS over time or can occur as a separate entity that never leads to structural changes in the axial skeleton [4]. It has been reported that, over a period of 2–10 years, progression to AS is seen in 10–40% of nr-axSpA patients [5]. Differences observed between the two subgroups include a higher male:female ratio (3:1 for AS [6] vs 1:1.6 for nr-axSpA [7]), higher levels of C-reactive protein (CRP) and lower rates of peripheral manifestations such as peripheral arthritis and enthesitis in AS compared with nr-axSpA [8, 9]. However, both subgroups present with similar clinical characteristics, have a similar burden of disease and are associated with substantially impaired physical function and health-related quality of life (HRQoL) [8–13]. Several studies have indicated that disease burden of AS and nr-axSpA are broadly similar to that of other inflammatory diseases, including psoriatic arthritis and rheumatoid arthritis [9, 14–17]. Disease onset typically occurs in early adulthood; given the chronic nature of the disease, patient HRQoL is substantially impaired [18]. Real-world data have shown significantly reduced HRQoL and work productivity in patients with nr-axSpA compared with general population controls [19].

HRQoL can be assessed using disease-specific patient-reported outcome (PRO) tools, such as the 18-item Ankylosing Spondylitis QoL (ASQoL) instrument [20]. During the development of the ASQoL, patients with AS were interviewed and respondents commented on the impact of pain and its effect on sleep, mood, motivation and ability to cope with everyday tasks, as well as the disease itself having major impacts on self-image, self-esteem and relationships [20]. The ASQoL has subsequently been widely used as a reliable instrument to assess the impact of AS and interventions on HRQoL from the patient perspective.

As many similarities are observed between AS and nr-axSpA patients in terms of disease characteristics, and studies have demonstrated content validity of AS PRO instruments in the broader axSpA population [21], the objective of this study was to explore the content relevance of the ASQoL instrument specifically in patients with nr-axSpA.

## Methods

### Study design and participants

This was an observational, cross-sectional, qualitative interview study conducted to evaluate the content validity of the ASQoL in a sample with nr-axSpA.

Patients were recruited from clinic settings, with efforts made to recruit a sample that varied by age, gender and race. Clinic staff used a screening form and checklist to review medical records and identify patients according to the eligibility criteria. Potential participants were contacted by telephone and, if interested in participating, were asked a short set of screening questions. Those considered eligible were invited for an enrollment visit; they were required to read, write, and speak English well enough to participate in the interview process.

In addition to recruiting patients from clinic settings, interviews from patients with axSpA who participated in a prior qualitative study were used [21]. The prior study was conducted to evaluate PRO instruments validated in AS in a broad axSpA population [21]. Interview transcripts from both groups of patients were analyzed in order to describe the symptoms and impacts experienced by patients and assess their relevance to the ASQoL items.

Patients interviewed for this study were ≥ 18 years of age with a documented diagnosis of adult-onset axSpA and met ASAS classification criteria for axSpA (excluding family history and good response to non-steroidal anti-inflammatory drugs) [22, 23]. Patients must have had inflammatory back pain for ≥ 12 months prior to screening and met either of the following definition criteria for nr-axSpA:

- active sacroiliitis (determined by MRI records in the patient’s medical history within the past year) plus ≥ 1 SpA feature (inflammatory back pain, Crohn’s disease, heel enthesitis, uveitis, psoriasis, elevated CRP, ulcerative colitis, arthritis, HLA-B27 or dactylitis)
- if sacroiliitis was not confirmed by MRI, CRP or erythrocyte sedimentation rate (ESR; Westergren scale) above the upper limit of normal (ULN; documented in medical records within the past month) plus presence of HLA-B27 and ≥ 1 other SpA feature.

Additional inclusion criteria were a Bath Ankylosing Spondylitis Disease Activity Index (BASDAI) score of ≥ 4 and a spinal pain score of ≥ 4 (BASDAI item 2, on a 0–10 numerical rating scale) [24]. Key exclusion criteria were presence of radiographic sacroiliitis, as defined by the mNY classification criteria (one radiographic criterion [bilateral Grade 2; unilateral Grade ≥ 3 on sacroiliac joint X-ray within 12 months of baseline] in addition to at least one of three clinical criteria) [3], and a diagnosis of any other type of inflammatory arthritis, fibromyalgia or any other secondary inflammatory condition, such as systemic lupus erythematosus.

Patients included in the prior qualitative analysis were aged ≥ 18 years with a diagnosis of axSpA of at least 3 months’ duration, as documented in their medical records [21]. To meet the specific diagnosis of nr-axSpA, patients were required to have active disease, defined as BASDAI score 4, spinal pain ≥ 4, CRP above the ULN or sacroiliitis on MRI as defined by ASAS criteria. Patients were excluded if they had a diagnosis of any other inflammatory arthritis or a known diagnosis of fibromyalgia.

The study was conducted in accordance with Good Clinical Practice and applicable regulatory requirements. Two clinic sites in the USA were involved in patient recruitment and enrollment; ethical approval of the study was provided centrally by Quorum Institutional Review Board. All patients provided written informed consent and completed a descriptive demographics form at the enrollment visit.

### Interview conduct and data collection

Patients interviewed for this study initially underwent a concept elicitation interview. This stage evaluated the relevance of the concepts (i.e., symptoms and impacts) measured by the ASQoL, whereby items that are important to patients are spontaneously elicited through open-ended interview questions. Semi-structured interview guides were developed to obtain feedback from patients relating to their symptom experiences, to identify specific language used by patients to express concepts related to nr-axSpA, and to provide structured rating exercises. Any other symptoms relating to nr-axSpA that the patient did not offer spontaneously were identified using follow-up probing questions and further description about each symptom was obtained. Symptom severity and symptom bothersomeness experienced by patients were elicited using an 11-point numerical rating scale, where 0 represented none and 10 represented extremely severe/bothersome. Impact difficulty was also rated on a scale of 0 (not difficult at all) to 10 (extremely difficult). Rating scale results were provided only for those symptoms and impacts expressed by patients.

After the concept elicitation interview, patients were given the ASQoL to complete. The ASQoL is self-administered, containing 18 items and taking up to 4 min to complete; items cover ability to perform activities of daily living, emotional impact, sleep/tiredness, and pain. A cognitive interview was then conducted to assess patients’ understanding of the ASQoL items, response options and instructions. The interviews were conducted by two different interviewers, each with experience in nr-axSpA and interview techniques for PRO development. Each interview was conducted in person in a private room at the clinic site. Interviews lasted approximately 90 min, and were audio-recorded and transcribed.

For the previously interviewed patients, interview transcripts were obtained but patients did not complete the ASQoL and, therefore, did not undergo cognitive interview.

### Sample size

Adequate sample sizes for qualitative research are based on reaching saturation of concept (the point at which no new information is being obtained). At this point, saturation of concept is considered to be achieved, indicating continued interviews with the same study population would be unlikely to yield new information and the resulting concept list derived from the interview process was considered sufficient [25, 26].

### Analysis of interview data

Two coders were involved in the coding process. A coding framework was developed to incorporate concepts outlined in the study goals and interview questions, which was revised via an iterative process as each transcript was coded [26]. The frequency with which a particular symptom or impact concept was expressed during the interviews, and the number of patients expressing it, were determined from the coded data as indicators of the relative predominance and importance of the concept to the patient sample.

The transcripts from the patients who had been interviewed previously (who did not complete the ASQoL or undergo the cognitive interview process) were also tabulated to identify expressions of the concepts contained in the ASQoL items; quotations were then lined up against the ASQoL items to show relevance of each concept to their symptom and impact experience.

Interview transcripts were ordered chronologically, based on interview completion date, and divided into four groups to identify the appearance of new symptoms and impact information, and assess saturation of concept. The codes that were derived from the second transcript group were compared with the codes that appeared in the transcripts from the first group. If new codes appeared in the second transcript group, it suggested that saturation had not been achieved and the comparison was repeated for the next group. This process was repeated for all four transcript groups.

Inter-rater agreement was used to assess the consistency of coding between the two coders. One of the content elicitation transcripts was independently dual coded and compared to evaluate any differences in code assignment between the two coders. The concept elicitation portions of all interview transcripts were coded for qualitative content analysis using ATLAS.ti software version 7.04, allowing concepts to be grouped by similar content and assessed for predominance [27].

Descriptive statistics were generated using SPSS statistics software (version 11.5) for all quantitative screening, demographic and rating data.

Overall, 18 patients participated in the study, including 13 patients for this study and five who were interviewed for a previous study. Patients’ median age was 46 years (range, 22–63) with the median age at diagnosis of chronic back pain being 29.5 years (range, 16–40); 89% were Caucasian and 67% were female (Table 1). Of the 18 patients, half had high school education only, while 28% had some college education. Evaluation of MRI records identified active (acute) inflammation highly suggestive of sacroiliitis associated with nr-axSpA in 14/18 patients and 13/18 patients were HLA-B27 positive. In addition to back pain, concurrent peripheral arthritis was reported by 10/18 patients, heel enthesitis by 4/18 patients, and uveitis, psoriasis or inflammatory bowel disease/Crohn’s disease/ulcerative colitis by 3/18 patients. Median time since diagnosis was 9.6 months (range, 3 months to 8 years) in the 13 new patients and 8.0 years (range, 3–15) in the five previously interviewed patients.

**Table 1:** Patient demographics and clinical characteristics

Characteristic	Total (*N* = 18)
Age (years)	
Median (range)	46.0 (22–63)
Gender	
Female	12 (67%)
Male	6 (33%)
Racial group	
White	16 (89%)
American Indian or Alaskan Native	1 (6%)
Black or African American	1 (6%)
Highest education level completed	
High school	9 (50%)
Some college	5 (28%)
Batchelor’s degree	3 (17%)
Graduate or Professional school	1 (6%)
Age when first diagnosed with chronic back pain	
Median (range)	29.5 (16–40)
Time since diagnosis (years)^a^	
Median (range)	0.8 (0.25–8.2)
Time since diagnosis (years)^b^	
Median (range)	8.0 (3.4–15.0)
Confirmed sacroiliitis, *n* (%)	
MRI	14 (78%)
Radiographic^b^	1 (6%)
Unconfirmed sacroiliitis, *n* (%)	
MRI negative but CRP or ESR > ULN^a^	2 (11%)
Other axSpA features, *n* (%)	
Inflammatory back pain	18 (100%)
HLA-B27 positive	13 (72%)
Arthritis	10 (56%)
Enthesitis (heel)	4 (22%)
Uveitis anterior	3 (17%)
Psoriasis	3 (17%)
Inflammatory bowel disease (Crohn’s disease/ulcerative colitis)	3 (17%)
Elevated CRP	9 (50%)
Dactylitis	0
BASDAI score at enrolment^a^	
Median (range)	7.2 (4.1–8.6)
Overall severity level of spondyloarthritis neck, back, or hip pain in past week (*0* = *none to 10* = *very severe*)^a^	
Median (range)	7.0 (6.0–8.5)

^a^Data for the 13 new patients only

^b^Data for the 5 previously interviewed patients only

### Concept elicitation interviews: data quality assessment

Seventy-eight percent of concepts offered by patients appeared in the first transcript group, 14% of new concepts appeared in the second transcript group, 5% in the third group, and 4% in the last four interviews (Online Resource 1), indicating that concept saturation had been reached. Two of the last three newly appearing codes were similar to others that had already been assigned in the energy/fatigue group and in the swelling group but used different descriptive terms and were in different physical locations. The final new code was for a patient who used the actual term ‘quality of life’. However, as the broad impact of a patient’s symptom experiences can be generalized to an impact on their quality of life, the single code created following analysis of this patient’s interview was covered by the overall picture provided by study data, and not considered a novel concept on its own even though the statement was coded as such to capture the general statement.

The inter-rater agreement resulted in 93% agreement between the two coders in the identification of a concept to code and 99% agreement in the assignment of codes.

### Signs and symptoms

Table 2 shows the percentages of patient expressions and the number of patients contributing those expressions to identify the overall predominance of the concepts that came forward in patient responses during the interview process. The most frequently expressed symptoms were related to pain (49% of all symptom expressions), specifically hip, neck general back, joint and spine pain (Table 2). The next most predominant symptoms mentioned by most patients were related to muscle contractions (stiffness), joint swelling, tenderness, tiredness, and insomnia (Table 2).

**Table 2:** Sign/symptom concepts and code frequencies

nr-axSpA symptom sub-domains and concepts	Total symptom concept expressions, *n* (%)	Transcripts contributing to concept expression, *n* (%)
Tiredness and fatigue	120 (12)	
Tiredness	50 (5)	15 (83)
Fatigue	34 (4)	11 (61)
Low energy	13 (1)	3 (17)
Weakness	13 (1)	5 (28)
Daytime sleepiness	6 (1)	4 (22)
Exhaustion	3 (0)	2 (11)
Lack of motivation	1 (0)	1 (6)
Sleep quality	78 (8)	
Trouble staying asleep	28 (39)	15 (83)
Sleep position limited	23 (2)	12 (67)
Trouble falling asleep	20 (2)	9 (50)
Poor sleep quality	7 (1)	3 (17)
Pain	481 (49)	
Joint pain	82 (8)	18 (100)
Hip pain	81 (8)	17 (94)
Low back pain	73 (7)	16 (89)
General back pain	70 (7)	16 (89)
Leg and foot pain	43 (4)	8 (44)
Neck pain	40 (4)	12 (67)
Unspecified pain	39 (4)	14 (78)
Spine pain	19 (2)	7 (39)
Chest pain	8 (1)	3 (17)
Arm pain	5 (1)	2 (11)
Mid back pain	5 (1)	2 (11)
Upper back pain	5 (1)	2 (11)
Diaphragm pain	4 (0)	1 (6)
Shooting pain	3 (0)	1 (6)
Rib pain	2 (0)	1 (6)
Pelvis pain	2 (0)	1 (6)
Swelling	49 (5)	
Joint swelling	37 (4)	13 (72)
Limb swelling	4 (0)	1 (6)
Diaphragm swelling	3 (0)	1 (6)
Low back swelling	3 (0)	1 (6)
Heel swelling	2 (0)	1 (6)
Muscle contractions	132 (13)	
Stiffness	102 (10)	18 (100)
Posture and stature changes	15 (2)	8 (44)
Muscle spasms	9 (1)	3 (17)
Tightness	6 (1)	4 (22)
Additional symptoms	120 (12)	
Tenderness	47 (5)	15 (83)
Joint popping	18 (2)	7 (39)
Other symptoms^a^	15 (2)	7 (39)
Numbness	14 (1)	4 (22)
Headache or migraine	10 (1)	4 (22)
Iritis	8 (1)	1 (6)
Restless legs	8 (1)	2 (11)

^a^Included bulging discs, constipation, feels ill, haemorrhoids, joint weakness, loss of muscle tone, muscle disconnected, paralysis, passing out, tendons pulling, and weight gain

During the concept elicitation interviews, patients spontaneously reported symptoms before probing questions were used to identify any other symptoms relating to nr-axSpA. The symptom concepts most often mentioned spontaneously by patients were general back pain (17/18 patients), stiffness (15/18 patients), hip pain (12/18 patients) and neck pain (10/18 patients) (Table 3).

**Table 3:** Symptoms and impacts reported spontaneously by patients and following probing

	Patients mentioning concept (*N* = 18)	
	Spontaneously reported, *n* (%)	Reported following probing, *n* (%)
Symptoms reported		
Tiredness/fatigue		
Tiredness	5 (28)	10 (56)
Fatigue	3 (17)	9 (50)
Insomnia/sleep quality		
Insomnia	2 (11)	11 (61)
Pain		
Hip	12 (67)	5 (28)
Neck	10 (56)	4 (22)
General back	17 (94)	
Joint	9 (50)	8 (44)
Spine	2 (11)	6 (33)
Leg and foot	5 (28)	
Chest	1 (6)	
Diaphragm	1 (6)	
Lower back	1 (6)	
Swelling		
Joint swelling	6 (33)	9 (50)
Muscle contraction		
Stiffness	15 (83)	3 (17)
Hand stiffness	1 (6)	
Additional symptoms		
Tenderness	4 (22)	12 (67)
Headache/migraine	1 (6)	
Iritis	1 (6)	
Joint popping	1 (6)	
Numbness	1 (6)	
Impacts reported		
Personal care (bathing, hygiene, dressing)	6 (33)	9 (50)
Trouble sleeping at night	13 (72)	4 (22)
Mobility		
Bending from waist, reaching overhead, rising	8 (44)	7 (39)
Standing unsupported	3 (17)	8 (44)
Climbing stairs unassisted	1 (6)	8 (44)
Looking over shoulder	1 (6)	8 (44)
Ability to do demanding physical activities	8 (44)	7 (39)
Doing daily activities (chores around house/garden, work)	9 (50)	6 (33)
Social relationships or activities	5 (28)	5 (28)
Transportation or travel restrictions	7 (39)	7 (39)
Maintaining relationships (friends and family)	4 (22)	8 (44)
Emotional impact (e.g., depression, frustration)	2 (11)	14 (78)
Self-confidence or self-image	2 (11)	6 (33)
Other		
Sexual activity affected	3 (17)	1 (6)
Constipation		1 (6)
Work impairment	1 (6)	

Symptom severity and bothersomeness ratings are shown in Table 4 for patients who reported having those specific symptoms as part of their experience. The most bothersome symptoms (median ≥ 8.0 on the numerical rating scale) were general back pain, low back pain, hip pain, neck pain and fatigue. The symptoms reported by at least 10 patients had median bothersome ratings of between 5.5 (tenderness and stiffness) and 8.0 (hip pain and neck pain). Of the most bothersome symptoms, the greatest symptom severity was general back pain and low back pain, both with a median rating of 9.0, and fatigue, with a median rating of 8.0.

**Table 4:** Symptom severity and bothersomeness ratings reported by patients (*N* = 18) scored on a scale of 0 to 10, with 0 being none and 10 being extremely severe/bothersome

Symptoms reported	Symptom severity		Symptom bothersomeness	
	*N*	Median (range)	*N*	Median (range)
Tiredness/fatigue				
Fatigue	7	8.0 (6–10)	9	8.0 (6–9)
Tiredness	11	6.0 (3–10)	12	7.5 (2–10)
Weakness	1	5.0	1	8.0
Insomnia and sleep quality				
Insomnia	3	7.0 (4–9)	4	4.5 (3–9)
Trouble falling asleep	1	10.0	2	4.5 (2–7)
Trouble staying asleep	1	6.0	3	3.0 (2–10)
Pain				
Arm pain	1	6.0	1	8.0
Chest pain	1	8.0	1	5.0
Diaphragm pain	1	10.0	1	8.0
General back pain	9	9.0 (5–10)	9	9.0 (6–10)
Low back pain	9	9.0 (6–10)	9	9.0 (5–10)
Hip pain	13	7.0 (6–10)	17	8.0 (5–10)
Joint pain	12	7.0 (5–9)	14	6.0 (3–10)
Leg and foot pain	10	8.0 (6–10)	11	7.0 (5–10)
Neck pain	13	7.0 (3–10)	13	8.0 (3–10)
Rib pain	1	4.0		
Spine pain	4	10.0 (5–10)	4	7.0 (5–10)
Swelling				
Joint swelling	6	5.0 (3–10)	11	6.0 (3–8)
Limb swelling	3	5.0 (3–7)	2	7.0 (6–8)
Muscle contractions				
Muscle spasm	1	10.0	1	7.0
Posture/stature			1	6.0
Stiffness	16	8.0 (3–10)	18	5.5 (3–10)
Back stiffness			1	6.0
Hand stiffness	2	4.5 (4–5)	2	5.5 (5–6)
Hip stiffness	1	4.0		
Neck stiffness	2	7.5 (7–8)	3	6.0 (6–8)
Additional symptoms				
Headache/migraine	2	6.0 (5–7)	2	8.5 (7–10)
Iritis	1	5.0	1	9.0
Joint popping	2	8.0 (8–8)	2	3.5 (2–5)
Numbness	1	6.0	1	6.0
Tenderness	13	6.0 (3–10)	14	5.5 (2–9)

Other symptoms with a median severity ≥ 7.0 and reported by more than 2 patients were spine pain, leg and foot pain, stiffness, neck stiffness, joint pain, and insomnia, with the latter being the least bothersome.

### Impact concepts

The impact-related concept of ‘needing assistance’ was reported by 17/18 patients and having their ‘walking impacted’ by 15/18 patients. Having ‘difficulty with personal care’ was reported by 14/18 patients and having ‘trouble bending’ by 13/14 patients. Twelve of the 18 patients reported having ‘trouble with stairs’ or having their ‘travel impacted’, 11 patients reported difficulty ‘rising from another position’, ‘difficulty getting around’, ‘difficulty performing general activities’ and ‘frustration’, or ‘coping behaviour’. Ten patients reported ‘difficulty standing’, ‘difficulty driving’, ‘difficulty performing housework and chores’, or having their ‘relationships affected’ (Online Resource 2).

The impact concept of ‘trouble sleeping at night’ was mentioned by 17/18 patients (Table 3). Emotional impacts were reported by 16 patients and 15 patients reported impacts to personal care or difficulty bending from the waist, reaching overhead, rising from chair or floor, ability to do demanding physical activity or doing daily activity. Transportation or travel restrictions were reported by 14 patients. It is interesting to note that emotional impact and, to a lesser extent, impact on personal care were mostly reported following probing whereas the other impacts, particularly impacts on sleeping, were either mostly spontaneously reported or more equally reported both spontaneously and by probing.

Impact difficulty ratings are shown in Table 5 for patients who reported each impact as part of their experience. The greatest difficulty was seen with doing general daily activities, playing sports, difficulty getting around, self-esteem, sexual activity, and worry/fear. With the exception of sexual activity, these impacts were only reported by 5 patients or fewer. Of the impacts reported by more than half the patients, the greatest difficulties related to doing daily activity around the home and bending.

**Table 5:** Impact difficulty ratings reported by patients (*N* = 18) on a scale of 0 to 10, with 0 being not difficult at all and 10 being extremely difficult

Impacts reported	Total patients (*N* = 18)	
	*N*	Median (range)
Physical activity limitations and restrictions		
Exercise	6	6.5 (5–9)
Sports	2	9 (8–10)
Walking	8	7.5 (4–10)
Restricted body movements		
Bending	10	6.5 (4–10)
Getting in car	1	4
Lifting/carrying	3	8 (4–9)
Rising	5	4 (2–10)
Sitting	6	8 (4–9)
Stairs	4	5 (3–8)
Standing	8	5 (3–9)
Turning	2	3.5 (3–4)
Difficulty getting around		
Driving	6	7 (4–10)
Getting around	1	9
Travel	8	7 (5–9)
Difficulty doing daily activity		
Child care	3	6 (4–10)
General	3	9 (9–9)
Home	11	7 (2–9)
Personal care	10	4 (2–7)
Work	8	8.5 (5–10)
Social/lifestyle limitations and restrictions		
Leisure	3	7 (6–9)
Relationships	7	8 (5–10)
Sexual activity	6	8.5 (7–10)
Social engagements	4	5 (3–8)
Emotional impacts		
Anger/irritations	3	7 (5–7)
Depression	3	7 (7–8)
Frustration	9	8 (4–10)
Self-esteem	4	8.5 (8–10)
Worry/fear	5	9 (6–9)
Aspects of burden		
Need assistance	2	8 (7–9)

### Cognitive interviews: data quality assessment

In the first wave of interviews (*n* = 5), all ASQoL items were understood and all patients were able to respond. Minor comments on the specific structure of some of the ASQoL items were made by patients who had been exposed to other questionnaires but all were able to clearly understand and provide meaningful responses to the ASQoL items. In the second wave of cognitive interviews (*n* = 4), the issues identified in the first round did not re-appear but one item’s response options were questioned (one patient had difficulty with only a yes or no response to item 6 [‘I am unable to join in activities with my friends/family’] and preferred to have a ‘sometimes’ response option). In the third wave of cognitive interviews (*n* = 5), none of the patients had difficulty choosing yes or no with the current response choices. While there were individual items that were not relevant to all patients, most patients were able to confirm that the items were relevant to their day-to-day experience of living with nr-axSpA.

### ASQoL content appropriateness

Quotations from the transcripts of the five previously interviewed patients were lined up against the items in the ASQoL to evaluate the relevance of ASQoL concepts to the patient experience. Although not every person had a quotation that could be matched to every concept, the overall coverage of concepts was robust and the concepts represented in the ASQoL items were relevant and important to the patient experience of nr-axSpA (Table 6).

**Table 6:** Example quotations from transcripts of the five previously interviewed patients across the ASQoL items

ASQoL item	Example concepts elicited from interviews
1. My condition limits the places I can go	*Can't go on long walks* *I cannot drive that long in a car any more* *It limits what I want to do that day, what I had planned*
2. I sometimes feel like crying	*It sucks. You want to cry* *The worst [pain]… where I'm in tears* *It is a deep aching to the point that I cry*
3. I have difficulty dressing	*It's hard to get dressed below the waist* *It's hard to try to put on your clothes to get ready* *Tying your shoes can be difficult sometimes*
4. I struggle to do jobs around the house	*I don't cook very often* *My house isn't as clean as what it used to be* *I am not always able to carry the laundry basket out to the line to hang them up*
5. It’s impossible to sleep	*I can’t sleep a full night* *Not getting restful deep sleep* *Just can't go to sleep*
6. I am unable to join in activities with my friends/family	*Not being able to do things I and my family want to do* *Can't be social with people* *I don't have a social life*
7. I am tired all the time	*I do feel tired a lot* *I'm always tired* *[I’m tired] every day*
8. I have to keep stopping what I am doing to rest	*Yes, [having to stop] depends on how long I've walked* *If I'm working on my tablet playing a game, I have to stop playing and move my position* *I took my daughters to the mall and we had to stop and rest several times while we were shopping*
9. I have unbearable pain	*There are times that's it's unbearable [low back pain]*
10. It takes a long time to get going in the morning	*Mornings are hard whenever I get up* *I get up in the morning and I’m really stiff, and then three, four hours later I feel better* *Getting yourself dressed takes a little bit longer [in the morning]*
11. I am unable to do jobs around the house	*I can't cut the grass anymore… I can't empty the bag or push mower anymore* *I can't even mop the floor*
12. I get tired easily	*I just feel tired all the time* *I could always use a nap; I do get very tired*
13. I often get frustrated	*Things that you can't do and others can* *I get frustrated very easily* *Frustrated, my temper gets the best of me*
14. The pain is always there	*Feels like almost a knife going into my back all the time* *Pain is constantly in that one area* *Dull ache, constant*
15. I feel I miss out on a lot	*You miss TV shows you like to watch* *I miss going to work because it kept me socialized with people* *I can't do a lot of things*
16. I find it difficult to wash my hair	*Bending over sometimes is an issue* *I can't get in the bathtub*
17. My condition gets me down	*I get depressed, you get tired of trying to explain to people how you feel* *I get irritable* *You see some people who are still capable of doing everything and it’s sad*
18. I worry about letting people down	*[I'm not] helping my kids with their schoolwork* *I worry about… not being enough for the kids and my husband* *Going somewhere with your kids, getting hurt, and having them see that*

## Discussion

As might be expected given the related underlying pathology, there are many similarities between AS and nr-axSpA; patient characteristics, burden of disease including impact on HRQoL and overall physical impairment, and relevance of PRO instruments have been found to be similar [8, 21, 28]. However, the relevance of the ASQoL content had not been explored previously in patients with nr-axSpA. The results of this qualitative study demonstrate that the similarities of the signs and symptoms and related impact on HRQoL experienced by these patient populations translates into a similarity in the appropriateness of ASQoL items.

There is currently a lack of well-designed qualitative research in patients with axSpA [21, 29, 30]. Our results obtained from face-to-face patient interviews add to the limited body of evidence in this disease setting. The concept elicitation interviews indicated that the concepts assessed by the ASQoL items were highly relevant to this patient population, accurately reflecting their experiences in terms of disease symptoms and the resulting impact on HRQoL. Furthermore, the results of cognitive interviews demonstrated that the ASQoL instructions, items and response scales were understood appropriately by patients, and related strongly to the symptoms and impacts raised in initial interviews. Finally, quotations obtained from five patients interviewed for a previous study, who did not complete the ASQoL, further substantiated the relevance of the concepts measured by the ASQoL items.

Our findings contribute to a growing body of tools available for the assessment of disease burden in patients with axSpA. It should be noted that, since this qualitative research was undertaken, the first AS-specific ASAS-HI has been developed; this is a health index rather than a HRQoL instrument, in that it provides an insight into whether problems are present in different categories of functioning rather than capturing the subjective experience of those problems [31]. The index aids assessment of overall functioning and health in patients with AS, and is a linear composite measure consisting of 17 items that cover most of the categories of the International Classification of Functioning, Disability and Health (ICF) core set. There is a degree of overlap between the ASQoL and ASAS-HI, with both PROs assessing key functional areas such as pain, sleep, tiredness, motivation, frustration and social interactions. However, there are some differences: the ASQoL assesses more emotional and domestic aspects of patients’ lives while the ASAS-HI has a strong emphasis on mobility and also includes financial and sexual components. It should also be noted that use of an established measure such as the ASQoL in a clinical research setting over newer tools such as ASAS-HI may be restricted by the terms of use; ASQoL has licensed status while ASAS-HI is freely available. This has an impact on the ability to gain a deeper understanding of how the ASQoL compares to newer generic/universal PROs.

The small sample size and the use of a convenience sample was a limitation of this study. Other limitations included the interview process being limited to the English language and the retrospective nature of some aspects of the study (X-rays were taken from existing medical records and detailed information on how the reviews were conducted was not available). The sample size and patient selection methodology for this study were consistent with this type of qualitative research, and results of concept saturation were satisfactory and indicated that a sufficient number of interviews had been conducted.

Evaluating the ASQoL in large cohorts of patients in the clinical trial setting would help to further validate its statistical performance in patients with nr-axSpA. RAPID-axSpA represents the first phase 3 study of certolizumab pegol involving patients with both nr-axSpA and AS to assess HRQoL measures, including ASQoL, across the axSpA disease spectrum (NCT01087762) [32]. More recently, data on the ASQoL as a secondary outcome measure in the recent C-AXSPAND phase 3, placebo-controlled study evaluating certolizumab pegol in patients with nr-axSpA can be useful to support these analyses (NCT02552212) [33].

Although sometimes under-estimated, nr-axSpA is frequently accompanied by severe symptoms and significant impairment in HRQoL. Validation of the ASQoL and other measures relevant to the axSpA disease spectrum will help to ensure that future clinical trials and cohort studies conducted in this disease setting are able to evaluate this impact on patients and provide a method of assessing change in HRQoL following therapy. In conclusion, to our knowledge, this is the first qualitative interview study conducted to evaluate the ASQoL in patients with nr-axSpA. Our study represents an important first step in confirming the relevance of the concepts included in the ASQoL in the nr-axSpA patient population, and demonstrates that the burden of nr-axSpA and impact on HRQoL is similar to patients with AS. Our results provide the basis for further quantitative assessment and psychometric analysis of the ASQoL to confirm its validity in patients with nr-axSpA. Once validated, this would confirm that the ASQoL represents a reliable, valid, and responsive endpoint for use in interventional clinical trials involving patients with nr-axSpA.

## Electronic supplementary material

Below is the link to the electronic supplementary material.

Electronic supplementary material 1 (DOCX 22 kb)

## Data Availability

Due to the small sample size in this trial, Individual Patient Data cannot be adequately anonymised and there is a reasonable likelihood that individual participants could be re-identified. For this reason, data from this trial cannot be shared.
