# Supplementary material for: Content validity of the ASQoL for use in a non-radiographic axial spondyloarthritis population: a qualitative study
Source: Qual Life Res. 2020 Jul 1;29(11):3155–66. doi: 10.1007/s11136-020-02552-z (PMC7591417; doi:10.1007/s11136-020-02552-z)
Supplement: Supplementary file 1 — Electronic supplementary material 1 (DOCX 22 kb) [file 11136_2020_2552_MOESM1_ESM.docx]

# Content validity of the ASQoL for use in a non-radiographic axial spondyloarthritis population

Mark C. Hwang,^1^ Mona Martin,^2^ Kristina Harris,^3^ Philip Geerdts,^4^ Jeffrey L. Stark,^5^ 
John Reveille^1^

^1^The University of Texas Health Science Center at Houston, McGovern Medical School, Houston, TX, USA

^2^Evidera PPD LLC, Bethesda, MD, USA

^3^UCB Pharma, Hong Kong, China

^4^UCB Pharma, Slough, UK

^5^UCB Pharma, Smyrna, GA, USA

**Journal:** Quality of Life Research

**Corresponding author email:** Mark.C.Hwang@uth.tmc.edu

**Online Resource 1.** Data saturation: number of concepts (Group 1) and new concepts (Groups 2–4). Saturation was calculated across transcript groups. Transcripts were grouped chronologically in the order the interviews were conducted.

| **Concept description** | **Group 1 (n=5 transcripts)** | **Group 2 (n=5 transcripts)** | **Group 3 (n=4 transcripts)** | **Group 4 (n=4 transcripts)** |
| --- | --- | --- | --- | --- |
| **Tiredness and fatigue** | | | | |
| Daytime sleepiness |  | X |  |  |
| Exhaustion |  | X |  |  |
| Fatigue | X |  |  |  |
| Lack of motivation |  |  |  | X |
| Low energy | X |  |  |  |
| Tiredness | X |  |  |  |
| Weakness | X |  |  |  |
| **Sleep quality** | | | | |
| Poor sleep quality | X |  |  |  |
| Sleep position limited | X |  |  |  |
| Trouble falling asleep | X |  |  |  |
| Trouble staying asleep | X |  |  |  |
| **Pain** | | | | |
| Arm pain |  | X |  |  |
| Low back pain | X |  |  |  |
| Mid back pain | X |  |  |  |
| Upper back pain | X |  |  |  |
| General back pain | X |  |  |  |
| Chest pain |  | X |  |  |
| Diaphragm pain |  | X |  |  |
| Hip pain | X |  |  |  |
| Joint pain | X |  |  |  |
| Leg or foot pain | X |  |  |  |
| Neck pain | X |  |  |  |
| Rib pain |  | X |  |  |
| Pelvis pain |  |  | X |  |
| Shooting pain |  |  | X |  |
| Spine pain | X |  |  |  |
| Unspecified pain | X |  |  |  |
| **Swelling** | | | | |
| Diaphragm swelling |  | X |  |  |
| Heel swelling |  | X |  |  |
| Joint swelling | X |  |  |  |
| Limb swelling | X |  |  |  |
| Low back swelling |  |  |  | X |
| **Muscle contraction** | | | | |
| Muscle spasms | X |  |  |  |
| Posture and stature changes | X |  |  |  |
| Stiffness | X |  |  |  |
| Tightness | X |  |  |  |
| **Additional symptoms** | | | | |
| Headache or migraine | X |  |  |  |
| Iritis | X |  |  |  |
| Joint popping | X |  |  |  |
| Numbness | X |  |  |  |
| Restless legs | X |  |  |  |
| Tenderness | X |  |  |  |
| **Physical activity limitations and restrictions** | | | | |
| Exercise impacted | X |  |  |  |
| Sports impacted | X |  |  |  |
| Walking impacted | X |  |  |  |
| **Restricted body movements** | | | | |
| Balance issues and falling | X |  |  |  |
| Trouble bending | X |  |  |  |
| Trouble getting in/out of a car |  | X |  |  |
| Hand mobility issues |  |  | X |  |
| Trouble lifting and carrying | X |  |  |  |
| Trouble lifting leg |  |  | X |  |
| Trouble reaching |  | X |  |  |
| Trouble rising from another position | X |  |  |  |
| Sitting impacted | X |  |  |  |
| Trouble with stairs | X |  |  |  |
| Trouble standing | X |  |  |  |
| Trouble turning and twisting | X |  |  |  |
| **Difficulty getting around** | | | | |
| Difficulty driving | X |  |  |  |
| Difficulty getting around | X |  |  |  |
| Travel impacted | X |  |  |  |
| **Difficulty doing daily activity** | | | | |
| Childcare impacted | X |  |  |  |
| Difficulty eating |  | X |  |  |
| Difficulty with housework and chores | X |  |  |  |
| Difficulty with general activities | X |  |  |  |
| Difficulty with personal care | X |  |  |  |
| Impacts on work | X |  |  |  |
| **Social and lifestyle limitations and restrictions** | | | | |
| Leisure activities impacted | X |  |  |  |
| Relationships affected | X |  |  |  |
| Sexual activity affected | X |  |  |  |
| Social activities affected | X |  |  |  |
| **Emotional impacts** | | | | |
| Anger or irritation | X |  |  |  |
| Anxiety | X |  |  |  |
| Depression | X |  |  |  |
| Frustration | X |  |  |  |
| Low self-esteem | X |  |  |  |
| Worry or fear | X |  |  |  |
| **Aspects of burden** | | | | |
| Needs assistance | X |  |  |  |
| **Coping behaviour** | | | | |
| Coping strategy | X |  |  |  |
| **Additional impacts** | | | | |
| Decreased quality of life |  |  |  | X |
| **Number (%) of concepts encoded in each group**  **(N=78)** | **61 (78.2)** | **11 (14.1)** | **4 (5.1)** | **3 (3.8)** |

**Online Resource 2.** Impact concepts and code frequencies

| **nr-axSpA symptom  sub‑domains and concepts** | **Total impact concept expressions, n (%)** | **Transcripts contributing to concept expression, n (%)** |
| --- | --- | --- |
| **Physical activity limitations and restrictions** | **74 (11.0)** |  |
| Walking impacted | 45 (6.8) | 15 (83.3) |
| Sports impacted | 16 (2.4) | 5 (27.8) |
| Exercise impacted | 12 (1.8) | 7 (38.9) |
| **Restricted body movements** | **154 (23.0)** |  |
| Sitting impacted | 28 (4.1) | 11 (61.1) |
| Trouble bending | 26 (3.8) | 13 (72.2) |
| Trouble lifting and carrying | 20 (2.9) | 9 (50.0) |
| Trouble rising from another position | 17 (2.5) | 11 (61.1) |
| Trouble with stairs | 16 (2.4) | 12 (66.7) |
| Trouble standing | 14 (2.1) | 10 (55.6) |
| Trouble turning and twisting | 10 (1.5) | 9 (50.0) |
| Balance issues and falling | 9 (1.3) | 5 (27.8) |
| Trouble getting in and out of car | 5 (0.7) | 3 (16.7) |
| Hand mobility issues | 3 (0.4) | 3 (16.7) |
| Trouble lifting leg | 3 (0.4) | 1 (5.6) |
| Trouble reaching | 2 (0.3) | 2 (11.1) |
| Other restricted body movements^a^ | 1 (0.1) | 1 (5.6) |
| **Difficulty getting around** | **78 (11.0)** |  |
| Difficulty driving | 33 (4.9) | 10 (55.6) |
| Travel impacted | 29 (4.3) | 12 (66.7) |
| Difficulty getting around | 16 (2.4) | 11 (61.1) |
| **Difficulty doing daily activity** | **131 (19.0)** |  |
| Difficulty with housework and chores | 41 (6.0) | 10 (55.6) |
| Difficulty with general activities | 36 (5.3) | 11 (61.1) |
| Difficulty with personal care | 27 (4.0) | 14 (77.8) |
| Impacts on work | 17 (2.5) | 9 (50.0) |
| Childcare impacted | 9 (1.3) | 5 (27.8) |
| Difficulty eating | 1 (0.1) | 1 (5.6) |
| **Social and lifestyle limitations and restrictions** | **79 (12.0)** |  |
| Social activities affected | 24 (3.5) | 9 (50.0) |
| Relationships affected | 23 (3.4) | 10 (55.6) |
| Leisure activities impacted | 18 (2.6) | 6 (33.3) |
| Sexual activity affected | 14 (2.1) | 6 (33.3) |
| **Emotional impacts** | **96 (14.0)** |  |
| Frustration | 28 (4.1) | 11 (61.1) |
| Anger or irritation | 16 (2.4) | 7 (38.9) |
| Low self-esteem | 16 (2.4) | 8 (44.4) |
| Worry or fear | 15 (2.2) | 7 (38.9) |
| Depression | 11 (1.6) | 7 (38.9) |
| Anxiety | 5 (0.7) | 3 (16.7) |
| Other emotional impacts^b^ | 5 (0.7) | 3 (16.7) |
| **Aspects of burden** | **20 (2.9)** |  |
| Needs assistance | 20 (2.9) | 17 (94.4) |
| **Coping behaviour** | **46 (6.8)** |  |
| Coping strategy | 46 (6.8) | 11 (61.1) |
| **Additional impacts** | **2 (0.3)** |  |
| Decreased quality of life | 2 (0.3) | 1 (5.6) |

^a^Included laying down; ^b^Included disgust, emotional instability, feels older and took a lot out of me
